# Frequency and predictors of concurrent complications in multi-suture release for syndromic craniosynostosis

**DOI:** 10.1007/s00381-023-06076-y

**Published:** 2023-07-18

**Authors:** Sujay Rajkumar, Daniel S. Ikeda, Michaela Scanlon, Margaret Shields, John R. Kestle, Jillian Plonsker, Michael Brandel, David D. Gonda, Michael Levy, Donald J. Lucas, Pamela M. Choi, Vijay M. Ravindra

**Affiliations:** 1 Drexel University School of Medicine, Philadelphia, PA USA; 2 Department of Neurosurgery, Walter Reed National Military Medical Center, Bethesda, MD USA; 3 Department of Neurosurgery, Naval Medical Center San Diego, San Diego, CA USA; 4 Division of Pediatric Neurosurgery, Primary Children’s Hospital, Salt Lake City, UT USA; 5 Department of Neurosurgery, Clinical Neurosciences Center, University of Utah, Salt Lake City, UT USA; 6 Department of Neurosurgery, University of California San Diego, San Diego, CA USA; 7 Division of Pediatric Neurosurgery, Rady Children’s Hospital, San Diego, CA USA; 8 Division of Pediatric Surgery, Department of General Surgery, Naval Medical Center San Diego, San Diego, CA USA

**Keywords:** Complication, Concurrent, Craniosynostosis, National Surgical Quality Improvement Program, Pediatric, Perioperative

## Abstract

**Purpose:**

Understanding the complication profile of craniosynostosis surgery is important, yet little is known about complication co-occurrence in syndromic children after multi-suture craniosynostosis surgery. We examined concurrent perioperative complications and predictive factors in this population.

**Methods:**

In this retrospective cohort study, children with syndromic diagnoses and multi-suture involvement who underwent craniosynostosis surgery in 2012–2020 were identified from the National Surgical Quality Improvement Program-Pediatric database. The primary outcome was concurrent complications; factors associated with concurrent complications were identified. Correlations between complications and patient outcomes were assessed.

**Results:**

Among 5,848 children identified, 161 children (2.75%) had concurrent complications: 129 (2.21%) experienced two complications and 32 (0.55%) experienced ≥ 3. The most frequent complication was bleeding/transfusion (69.53%). The most common concurrent complications were transfusion/superficial infection (27.95%) and transfusion/deep incisional infection (13.04%) or transfusion/sepsis (13.04%). Two cardiac factors (major cardiac risk factors (odds ratio (OR) 3.50 [1.92–6.38]) and previous cardiac surgery (OR 4.87 [2.36–10.04])), two pulmonary factors (preoperative ventilator dependence (OR 3.27 [1.16–9.21]) and structural pulmonary/airway abnormalities (OR 2.89 [2.05–4.08])), and preoperative nutritional support (OR 4.05 [2.34–7.01]) were independently associated with concurrent complications. Children who received blood transfusion had higher odds of deep surgical site infection (OR 4.62 [1.08–19.73]; p = 0.04).

**Conclusions:**

Our results indicate that several cardiac and pulmonary risk factors, along with preoperative nutritional support, were independently associated with concurrent complications but procedural factors were not. This information can help inform presurgical counseling and preoperative risk stratification in this population.

## Introduction

Craniosynostosis occurs in nearly 1 in 2000–2500 live births [1, 2]. Because craniosynostosis is associated with learning impairment, headaches, and potential visual loss and blindness [3], cranial vault remodeling (CVR) is performed to expand the cranium and recontour the abnormal head shape [4]. Although advancements in pediatric anesthetic care and surgical techniques have reduced the incidence of severe complications [5], CVR remains associated with complications such as brain injury, airway problems, acute blood loss anemia, and potentially death [6, 7].

Most cases of craniosynostosis involve a single suture, but multiple sutures can be involved. Craniosynostosis can also occur in children with any of over 130 different syndromes. A reported 7–72% of patients with craniosynostosis have concurrent congenital neurological, cardiac, renal, and other body system-related anomalies [8–10]. Because of the complexity of syndromic-related craniosynostosis, consideration should be given to complications that occur after surgery, especially if they warrant readmission; in addition to previously listed indications, children with syndromic craniosynostosis often require corrective maneuvers for midface hypoplasia for airway difficulty. A comprehensive assessment of factors that adversely affect a patient’s recovery (independently or concurrently) after craniosynostosis is warranted. There are no studies examining co-occurring complications among pediatric patients with syndromic diagnoses undergoing multi-suture craniosynostosis surgery.

Specifically in young children with multi-suture involvement, it not known which complications cluster after craniosynostosis correction. To stratify risk and understand complication profiles, we examined whether relationships exist among complications and specifically whether there is complication clustering in children with syndromic conditions. By using a large U.S. surgical quality database, we analyzed rates of independent and concurrent complications after craniosynostosis surgery in children with syndromic diagnoses. We also assessed factors associated with concurrent postoperative complications to help with risk stratification. We hypothesized that certain patient- and procedure-related factors predispose children with syndromic diagnoses undergoing multi-suture release to concurrent complications.

## Methods

### Data source

This retrospective cohort study used the American College of Surgeons (ACS) National Surgical Quality Improvement Program-Pediatric (NSQIP-P) database. NSQIP-P includes 30-day perioperative and surgical outcomes for individuals ≤ 18 years. Variables are collected from > 60 pediatric institutions on an 8-day systematic sampling cycle that allows for proportional diversity in selection [11]. The NSQIP-P data are audited and validated continually and organized into Participant User Files (PUFs). For the current investigation, the 2012–2020 data were used [12]. The use of ICD-9 and ICD-10 codes was based on the method of coding in the NSQIP-P records during the inclusion years of the study (2012–20). Because NSQIP-P PUF datasets do not have patient identifying information, the study was exempt from institutional ethics review.

### Study design and inclusion criteria

Patients ≤ 3 years of age with a syndromic diagnosis who underwent multi-suture craniosynostosis release and other cranial reconstruction procedures were included. The classification system for diagnoses of “congenital malformations” in the NSQIP-P was the International Classification of Diseases-9 and for procedures was the Current Procedural Terminology (CPT). The included diagnoses were Apert, Pfeiffer, or Saethre-Chotzen syndrome (ICD-9 code 755.55), craniofrontal dysplasia (759.81), Carpenter syndrome (759.89), and Crouzon’s/other congenital and musculoskeletal anomalies (756). The included procedures were craniotomy for craniosynostosis—frontal or parietal bone flap and bifrontal bone flap (CPT codes 61556 and 61557); extensive craniectomy for multiple suture craniosynostosis, not requiring and requiring bone grafts (61558 and 61559); reconstruction, bifrontal, superior-lateral orbital rims and lower forehead, advancement or alteration (e.g., plagiocephaly, trigonocephaly, brachycephaly), with or without grafts (includes obtaining autografts) (21175); and repair, revision, and/or reconstruction procedures on the head (21180). Only primary CPT codes were used for identification. Patients who underwent emergent surgery or had prior surgery within 30 days were excluded from the analysis.

The primary outcome was concurrent complications (≥ 2 complications occurring intra- or perioperatively up to 30 days affecting multiple organ systems). Secondary outcomes included non-home discharge, days of mechanical ventilation, readmission, length of stay, and death.

### Statistical analysis

Frequencies of each complication were calculated, cross-tabulated, and ranked to identify the most frequent concurrent complications.

A random forest analysis was performed using Python (v1.1.2, https://www.python.org/) to identify patient and treatment factors associated with the risk of concurrent complications. Top factors were identified, and binary logistic regression was performed to identify odds ratios (ORs) for the most influential factors for the primary outcome. The comparison group was children with syndromic disease who experienced zero or one complication.

Correlation analyses were used to identify relationships between the number of perioperative complications and the secondary outcomes. Because of the high rate of blood transfusion, univariate logistic regression was performed to identify the odds of having any complication when the patient received blood transfusion.

Descriptive and logistic regression analyses were performed using the SPSS statistical software (v26). A p-value of < 0.05 was considered statistically significant. The STROBE checklist was used for study reporting.

## Results

### Cohort overview

**Fig. 1:**
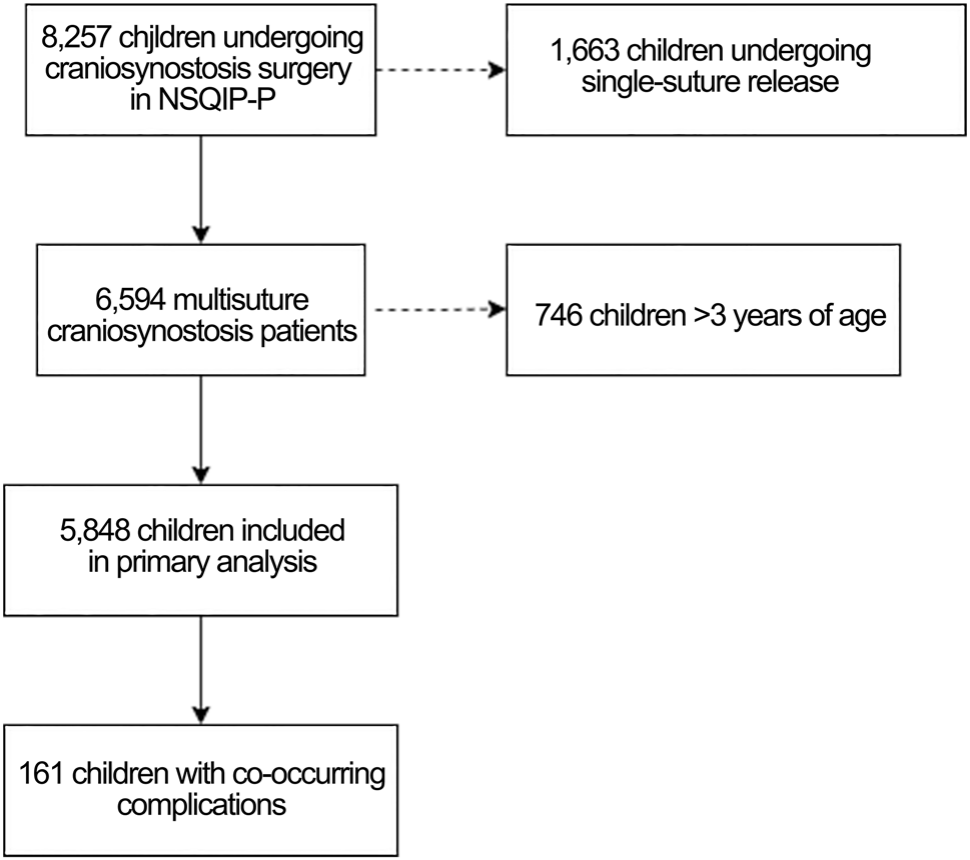
Flow diagram indicating inclusion in the study

**Fig. 2:**
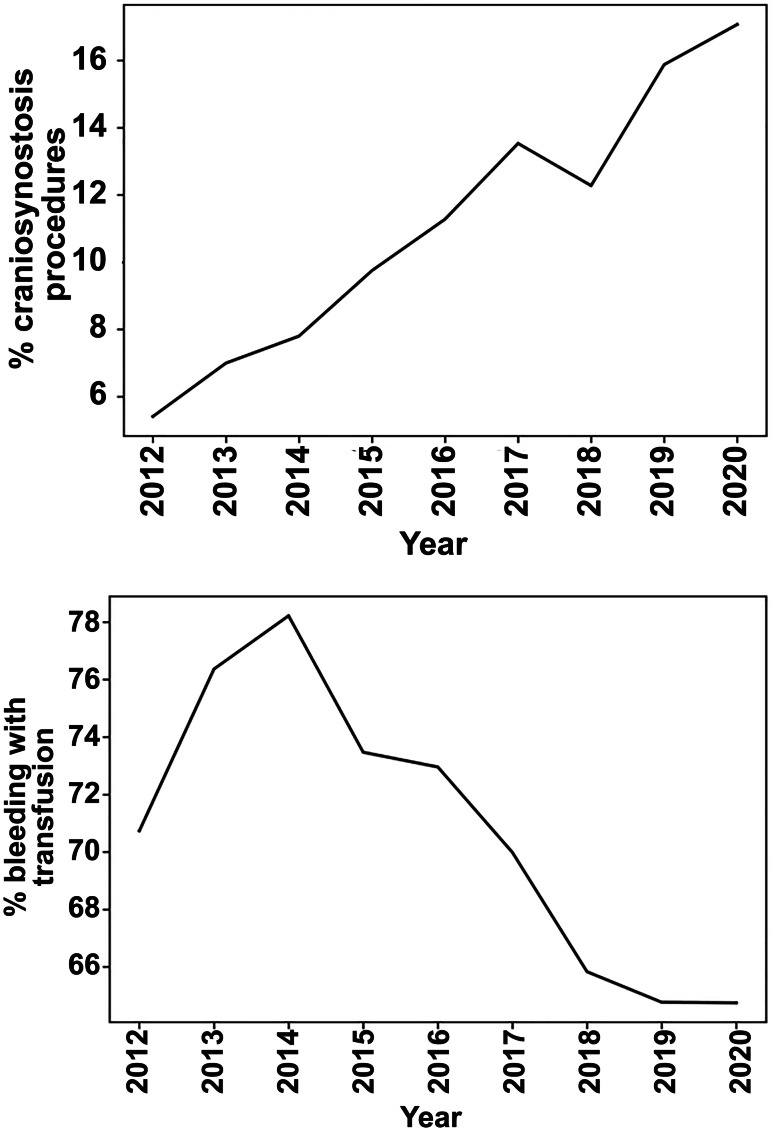
(Top) Frequency of craniosynostosis procedures by year as a percentage of all operations from 2012 through 2020. (Bottom) Rates of bleeding with subsequent transfusion in patients undergoing multi-suture cranial vault remodeling, by year from 2012 through 2020

A total of 5,848 children who underwent multi-suture synostosis surgery were included (Fig. 1). The frequency of procedures steadily increased over time (Fig. 2, top). The median age of the entire cohort was 0.69 years [interquartile range (IQR) 0.43–1.00], and 3771 (64.48%) of children were male. Most children were white (4215, 72.08%), and 4158 (71.10%) had a body mass index < 18.5 kg/m^2^ (Table 1).

**Table 1:** Baseline demographic, clinical, and surgical variables for children undergoing multi-suture craniosynostosis repair (n = 5,848)

**Variable**	**Value**^a^
Median age (years)	0.69 (IQR: 0.43–1.00)
Female	2077 (35.52%)
Race	
White Black Asian American Indian or Alaskan Native Native Hawaiian/Pacific Islander Other Unknown	4215 (72.08%) 468 (8.00%) 130 (2.22%) 53 (0.91%) 10 (0.17%) 8 (0.14%) 964 (16.48%)
BMI	
Underweight Normal Overweight Obese	4158 (71.10%) 1626 (27.80%) 36 (0.62%) 28 (0.48%)
Impaired cognitive status at time of surgery	698 (11.94%)
Nutritional support at time of surgery	226 (3.86%)
Oxygen support at time of operation	68 (1.16%)
Ventilator dependence	48 (0.82%)
History of asthma	116 (1.98%)
History of bronchopulmonary disorder	161 (2.75%)
Gastrointestinal disease	497 (8.50%)
Cardiac risk factors	
None Minor Major Severe	5248 (89.74%) 372 (6.36%) 213 (3.64%) 15 (0.26%)
Seizure disorder	77 (1.32%)
Cerebral palsy	25 (0.43%)
Structural CNS abnormality	1236 (21.14%)
Hematologic disorder	151 (2.58%)
Inotropic support at time of operation	32 (0.55%)
ASA status^b^	
I II ≥ III	991 (16.95%) 3395 (58.05%) 1458 (24.93%)
Procedure	
Reconstruction, bifrontal, superior-lateral orbital rims and lower forehead, advancement or alteration (CPT 21175)	1420 (24.28%)
Repair, revision, and/or reconstruction procedures on the head (CPT 21180)	33 (0.56%)
Craniectomy for craniosynostosis (CPT 61556)	254 (4.34%)
Craniotomy for craniosynostosis; bifrontal bone flap (CPT 61557)	562 (9.61%)
Extensive craniectomy for multiple cranial suture craniosynostosis; not requiring bone grafts (CPT 61558)	366 (6.26%)
Extensive craniectomy for multiple cranial suture craniosynostosis; requiring bone grafts (CPT 61559)	3213 (54.94%)
Syndromic diagnosis^c^	
Crouzon/other syndromic disease (ICD-9 756) Apert, Pfeiffer, Saethre-Chotzen syndromes (ICD-9 755.55) Craniofrontal dysplasia (ICD-9 759.81) Carpenter syndrome (ICD-9 759.89)	5839 (99.84%) 13 (0.22%) 1 (0.02%) 29 (0.50%)
Median total operation time (min)	186 (IQR: 116–262)

*IQR* interquartile range, *BMI* body mass index, *CNS* central nervous system, *AS* American Society of Anesthesiologists, *CPT* Common Procedural Terminology

^a^Values reported as number (%) unless otherwise indicated

^b^Four without ASA classification assigned

^c^Patients may have concurrent syndromic diagnoses

Nearly 700 (11.94%) children were documented as having impaired cognitive status at time of surgery, and 226 (3.86%) children were on nutritional support. Oxygen support was present in 68 (1.16%) children and 48 (0.82%) children were ventilator dependent at the time of surgery. Nearly 2% of children (116) had a history of asthma and 161 (2.75%) had a bronchopulmonary disorder; 497 (8.50%) had a history of gastrointestinal disease. Cardiac risk factors were present in ~ 10% of children. Additionally, 77 (1.32%) children had a seizure disorder, 1236 (21.14%) were diagnosed with a structural central nervous system abnormality, 151 (2.58%) had a hematologic disorder, and 32 (0.55%) were on inotropic support at the time of surgery.

The most common procedures were extensive craniectomy for multiple cranial suture craniosynostosis requiring bone grafts (CPT 61559) and bifrontal reconstruction of superior-lateral orbital rims and lower forehead, advancement or alteration (CPT 21175) (approximately 80% of cases). Most (99.84%) patients were coded as Crouzon’s/other syndromic disease (ICD-9 756).

### Outcomes

The median length of hospital stay was 5 days [IQR 4–7 days] (Table 2), with 22 (0.38%) requiring > 30 days in the hospital. After surgery, the mean number of days on mechanical ventilation was 0.18 ± 1.81, but 5 patients (0.09%) required > 30 days of mechanical ventilation. At discharge, 5,802 (99.21%) patients went home. One child (0.02%) died before discharge, another died within 30 days of surgery, and 17 (0.29%) were unaccounted for.

**Table 2:** Outcomes for children undergoing multi-suture craniosynostosis repair (n = 5,848)

**Outcome**	**Value**^a^
Perioperative complications	4,107 (70.23)
Concurrent complications	161 (2.75)
Median length of hospital stay (days)	5 (IQR: 4–7)
Hospitalization > 30 days	22 (0.38)
Mean days on mechanical ventilation (± SD)	0.18 ± 1.81
> 30 days on mechanical ventilation	5 (0.09)
Home discharge	5,802 (99.21)
Non-home discharge	29 (0.50)
Discharge destination unknown	17 (0.29)
Readmission within 30 days	184 (3.15)
Death within 30 days	2 (0.03)

*SD* standard deviation

^a^Values reported as number (%) unless otherwise indicated

### Complication overview

Among the 5,848 patients, 1741 (29.77%) did not experience a complication. The overall perioperative complication rate was 70.23% (4107/5848), and the overall concurrent complication rate (≥ 2) was 2.75% (161/5848). Stratified by number of complications, 3946 (67.48%) children experienced one complication and 129 (2.21%), 26 (0.44%), and 6 (0.11%) experienced two, three, and four or more complications, respectively. Table 3 demonstrates the frequency of specific complications. The most common complication was hematologic, with 69.53% of all patients requiring transfusion, despite the overall incidence of blood transfusion steadily decreasing from 2012 to 2020 (Fig. 2, bottom). There were 244 nonhematologic complications, of which superficial infection/surgical site infection was the most prevalent (1.08%).

**Table 3:** Complications among multi-suture craniosynostosis surgery patients

**Complication**	**Number (%)**
Bleeding/transfusion	4,066 (69.53)
Urinary tract infection	8 (0.14)
Pneumonia	11(0.19)
Venous thrombosis	5 (0.09)
Superficial infection/SSI	63 (1.08)
Deep wound infection/SSI	24 (0.41)
Organ/space infection	21 (0.36)
Wound disturbance/dehiscence	17 (0.29)
Unplanned reintubation	22 (0.38)
Renal insufficiency	0
Renal failure	0
Coma	1 (0.02)
CVA/stroke or intracranial hemorrhage	5 (0.09)
Seizure	22 (0.38)
Nerve injury	0
Cardiac arrest	9 (0.15)
*C. diff* infection	6 (0.10)
Sepsis	24 (0.41)
Septic shock	2 (0.03)
Central line–associated bloodstream infection	2 (0.03)
Death within 30 days	2 (0.03)

*SSI* surgical site infection, *CVA* cardiovascular accident, *C. diff Clostridium difficile*

### Complication co-occurrences

One hundred sixty-one children (2.75%) experienced > 1 complication. When assessing complications by body system, 82% of concurrent complications were hematologic/infectious and 59% hematologic/wound-related. Table 4 demonstrates the most frequently concurrent complications, which were led by transfusion/superficial infection (27.95%), transfusion/deep incisional surgical site infection (13.04%), and transfusion/sepsis (13.04%). The top 10 concurrent complications all included bleeding with subsequent transfusion. The most frequent concurrent complications excluding bleeding/transfusion were site-specific/systemic infections (5 children, 3.11%), cerebrovascular accident/seizure (4 children, 2.48%), and infection/wound disturbance (4 children, 2.48%).

**Table 4:** Co-occurring complication frequency among multi-suture craniosynostosis surgery patients

**Co-occurring complications (Total = 161)**	**Number (%)**
Bleeding/transfusion & superficial infection	45 (27.95)
Bleeding/transfusion & deep incisional SSI	21 (13.04)
Bleeding/transfusion & sepsis	21 (13.04)
Bleeding/transfusion & unplanned reintubation	19 (11.80)
Bleeding/transfusion & seizure	17 (10.56)
Bleeding/transfusion & organ/space SSI	16 (9.94)
Bleeding/transfusion & wound disturbance/dehiscence	13 (8.07)
Bleeding/transfusion & urinary tract infection	8 (4.97)
Bleeding/transfusion & pneumonia	8 (4.97)
Bleeding/transfusion & cardiac arrest	6 (3.73)

### Factors associated with concurrent complications

Six statistically significant factors were associated with the outcome of concurrent complications (Table 5), including five that increased the risk of concurrent complications. Two cardiac-related factors were elucidated: major cardiac risk factors (OR 3.50 [95% CI 1.92–6.38]) and previous cardiac surgery (OR 4.87 [2.36–10.04]). Two pulmonary factors were also identified: preoperative ventilator dependence (OR 3.27 [1.16–9.21]) and structural pulmonary/airway abnormalities (OR 2.67 [1.27–5.63]). Finally, an additional risk factor was preoperative nutritional support (OR 4.05 [2.34–7.01]). Non-premature birth was found to be protective against co-occurring complications (OR 0.48 [0.35–0.67]). No surgical treatment factors were identified as significant risk factors associated with concurrent complications.

**Table 5:** Treatment/patient characteristics associated with co-occurring complications among multi-suture craniosynostosis surgery patients

**Treatment/patient characteristics**	**Odds ratio (95% CI)**^b^	**p value**
**Major cardiac risk factors**	**3.50 (1.92–6.38)**	**< 0.001**
Elevated preoperative white blood cell count	1.95 (0.81–4.73)	0.14
**Ventilator dependence**	**3.27 (1.16–9.21)**	**0.03**
**Preoperative nutritional support**	**4.05 (2.34–7.01)**	**< 0.001**
**Structural pulmonary/airway abnormalities**	**2.67 (1.27–5.63)**	**0.01**
**Neonate (No)**	**0.48 (0.35–0.67)**	**< 0.001**
Female	0.98 (0.70–1.38)	0.92
Tracheostomy	2.31 (0.87–6.13)	0.09
Elevated preoperative INR	3.66 (0.73–18.28)	0.11
**Previous cardiac surgery**	**4.87 (2.36–10.04)**	**< 0.001**

Boldface values indicate statistical significance at *p* < 0.05

^a^Ranked as identified by random forest analysis

^b^Binary logistic regression was used to determine odds ratios of complication occurrence using the non-concurrent complication group as a reference

### Concurrent complications and correlations with clinical outcomes

Having a greater number of concurrent complications weakly correlated with hospital readmission (r = 0.25; p < 0.001). No correlation existed between number of concurrent complications and total hospital stay, number of ventilation days, non-home discharge, re-admission, and death (Table 6). Given the prevalence of transfusion, further analysis revealed blood transfusion was associated with higher risk of deep incisional surgical site infection (OR 4.62 [1.08–19.73] (p = 0.04).

**Table 6:** Correlation between the number of co-occurring complications and secondary outcomes

**Outcome variable**	**Correlation coefficient (r)**	**p value**
Total hospital stay (days)	0.05	< 0.001
Days ventilation	0.09	< 0.001
Non-home discharge	0.03	0.02
Readmission	0.25	< 0.001
Death	0.04	< 0.001

## Discussion

Craniosynostosis surgery is often performed to address elevated intracranial pressure and severe cosmetic deformity; thus, procedures can be urgent but are performed electively and require planning. Because the frequency of craniosynostosis surgery is increasing, understanding which children are at risk for complications, and specifically multiple complications, is critical. In the study population, concurrent complications occurred in 2.75% of children with syndromic disease who underwent suture release. We identified multiple risk factors associated with concurrent complications.

### Bleeding/transfusion

We found an overall complication rate of 70.23%, which reduced to 30% when bleeding requiring transfusion was excluded, which is significantly higher than reported previously [13]. Perioperative bleeding is significant during craniosynostosis procedures and comprised 94% of all complications in this study because blood transfusion is considered a complication in the NSQIP-P data. Allogenic blood is commonly transfused during CVR, with some institutions reporting transfusion rates as high as 100% [14]. Although some argue that blood transfusion is “expected,” we argue that transfusion should be considered a complication because of the potential consequences of the intervention. Interestingly, the rate of blood transfusion is decreasing over time, reflecting an improvement in surgical technique, attention to blood loss, and methods to reduce intraoperative bleeding.

Six of the most common concurrent complications identified in this study were hematologic/infectious, suggesting a possible relationship between bleeding requiring transfusion and perioperative infection. We found that blood transfusion was associated with higher risk of deep incisional surgical site infection (OR 4.62 [1.08–19.73]. This is similar to a meta-analysis of 23 prospective controlled clinical trials, which observed that postoperative allogenic blood transfusions across various procedures were associated with a significantly increased odds of postoperative bacterial infections (common OR 5.26 [5.03–5.43]) [15]. The specific causative factor of this association remains unclear; however, it is evident that allogeneic blood leads to a greater degree of postoperative immunosuppression than autologous transfusions or no transfusion. Therefore, it is compelling to augment intraoperative maneuvers with the aim of reducing bleeding/transfusions and subsequent infectious complications as well. A 2022 study suggested that the use of tranexamic acid in a dose-independent manner for craniosynostosis procedures reduced the rates of perioperative bleeding and transfusion [16]. Another study reported that maintaining normothermia during craniosynostosis repair, requiring pretransfusion surgical consultation, and using a preincisional bolus of tranexamic acid and incisional lidocaine with epinephrine reduced the institutional transfusion rate from 100% to 22.7% over 5 years [17]. Institutional adoption of clinical guidelines for anesthesia and surgery may explain the proportional decrease in bleeding/transfusions observed in our study.

### Nonhematologic complications

Nonhematologic complication rates vary in the literature because of the differing data sources and classification schemes chosen by authors. One small retrospective review observed a postoperative complication rate of 2.86% (one patient experiencing wire protrusion) when bleeding/transfusion was excluded [18]. Despite a reoperation rate of 8.6%, the authors concluded that open craniosynostosis surgery can be performed with minimal complications, low recurrence rates, and satisfactory cosmetic outcomes. A higher complication rate (35.9%) was reported by Shastin et al. [19] using a larger sample size and including only “excessive” bleeding/transfusion as a complication. The inclusion of complications > 30 days in both inpatient and outpatient settings may have contributed to their higher reported rate. A 2012 study comparing complication rates from the NSQIP-P and the Kids’ Inpatient Database (KID) described rates excluding blood transfusion at 2.9% and 3.0%, respectively [20]. For comparison, our nonhematologic complication rate was 4.17%, which is likely higher because we included only children with syndromic diagnoses. Future studies should focus on postoperative complication rates by using common data elements and standardized definitions.

### Risk factors

Five clinical factors were identified as having a statistically significant association with increased risk of concurrent complications. These were major cardiac risk factors, previous cardiac surgery, preoperative ventilator dependence, structural pulmonary/airway abnormalities, and preoperative nutritional support.

#### Cardiac risk factors

Previous explorations of the relationship between cardiac disease in children with craniosynostoses and postoperative complications has been inconclusive. Using NSQIP-P 2012–2016 PUFs, Bartz-Kurycki et al. [21] independently observed an association between cardiac risk factors and complications, but that relationship failed to remain significant upon multivariate analysis. Another study using NSQIP-P to identify factors associated with reoperation in craniosynostosis patients (2012–2014) also failed to identify a relationship [9]. Conversely, Parikh et al. [22] found that cardiac disease was an independent risk factor for complications (p < 0.01) when using NSQIP-P. Thus, further analysis of the relationship between cardiac factors and complications is critical.

#### Structural/pulmonary abnormalities

Although there is no direct relationship between structural pulmonary abnormalities and premature cranial suture fusion, certain conditions can affect both the lungs and the skull. Children with Apert syndrome can have significant airway narrowing and abnormal lung development [23]. Syndromic craniosynostoses are also frequently associated with obstructive sleep apnea, often due to midface hypoplasia [24]. Furthermore, up to 40% of children with syndromic craniosynostosis were shown to have laryngotracheal anomalies such as subglottic stenosis, tracheal stenosis, laryngeal cleft, laryngeal webs, and tracheomalacia [25]. Thus, children with syndromic craniosynostosis, particularly those with diagnosed structural pulmonary abnormalities or who are ventilator dependent, are at greater risk for respiratory complications during and after surgery.

#### Preoperative nutritional support

Although we found preoperative nutritional support was associated with co-occurring complications, this may represent inherent risk of the study population, rather than a true “modifiable” risk factor. Pereira et al. [26] found that feeding and nutrition were present in a small cohort of children with Apert syndrome. In parallel to the current findings, Sherrod et al. [27] broadly demonstrated that nutritional support was an independent risk factor (OR 1.4) for unplanned readmission after pediatric neurosurgery procedures. For children on nutritional support, it may be prudent to delay surgery, if possible, to minimize their risk of complications.

#### Patient age

Our study demonstrated that children born at term had a lower risk of co-occurring complications, likely secondary to overall general health and lack of comorbid conditions associated with prematurity. Previous studies had demonstrated that greater age at surgery was a risk factor for complications after craniosynostosis procedures. Using the KID, Bruce et al. [28] observed that procedures performed after 12 months of age carried higher odds of perioperative complications than procedures performed before 12 months (OR 1.61 [1.27–2.06]). For children > 36 months, the odds increased to 2.53 [1.67–3.82]. In addition to having fewer perioperative complications, Patel et al. [29] described that patients who undergo CVR at a younger age typically have better long-term psychiatric, neurologic, and intellectual outcomes. Our model did not identify age as a risk factor, likely because of the strict inclusion of children under 3 years. Age differed among reported outcomes between the KID and NSQIP-P database [20], which may explain some of the difference between our study and that of Bruce et al. Importantly, our model did not identify any procedural factors such as intraoperative time or type of procedure as predictive factors of concurrent complications.

### Limitations

There are limitations to this investigation. The NSQIP-P database is retrospective, and although craniosynostosis procedures are captured, the database was not built specifically for these patients. However, NSQIP-P accurately captures patient characteristics, treatment factors, and early postoperative complications because of the ACS’s strict data integrity requirements for participating hospitals and trained reviewers. In NSQIP-P, there is no detailed information regarding the severity of deformity and correction, procedural approaches, or surgical techniques. Although outdated, the use of ICD-9 and ICD-10 codes was based on coding methods used in the database during the inclusion years of the study (2012–20). The use of CPT codes is the most accurate way to search and define surgical treatment using the NSQIP-P data. This may limit the global applicability of this study, but there is precedence for a study like this [30] and currently over 60 countries use CPT terminology to date [31].

The procedural codes used here are a modest proxy for risk and severity, although indirect. There is substantial difference in procedural morbidity in syndromic synostosis procedures depending on the indication and specific procedure (often encompassed under the same CPT code) and expected associated complications. It is difficult to parse out complications by procedure because of this heterogeneity, which limits the extrapolation of the results. In addition, 99% of the diagnoses were Crouzon/other syndromic disease (ICD-9 756), which encompasses a wide array of conditions, although using a national administrative database is useful for studying conditions with low incidence and low complication rates.

For this study, we chose to include children ≤ 3 years to consider only primary procedures and prohibit inclusion of older children at higher risk for complications. Although institutional thresholds for blood transfusion are often present, they are not standardized among NSQIP-P centers. Furthermore, it is unclear how NSQIP-P defines developmental delay for infants, making it difficult to ascertain its significance. Furthermore, the NSQIP-P database is based on data collected up to 30 days postoperatively so longer-term complications and concurrent complications are not captured. Finally, we would caution international generalizability with respect to the codes used for study inclusion as the cohort was derived from a US sample.

## Conclusions

This is the first study to examine concurrent complications by body system and complication type in children ≤ 3 years of age with syndromic diagnoses undergoing multi-suture craniosynostosis surgery. The concurrent complication rate was 2.75%. The most common concurrent complications were transfusion/superficial infection (28%) followed by transfusion/deep incisional surgical site infection (13%) and transfusion/sepsis (13%). Major cardiac risk factors, previous cardiac surgery, preoperative ventilator dependence, structural/pulmonary abnormalities, and preoperative nutritional support were the baseline factors associated with increased risk of concurrent complications; no procedural factors were found as predictors of concurrent complications. This information can inform presurgical counseling and multidisciplinary planning by focusing on high-risk children to improve surgical outcomes.

## Data Availability

NSQIP-P data are from a publicly obtainable data source by purchase/involvement as a site.

## Declarations

### Copyright statement

“I am a military Service member. This work was prepared as part of my official duties. Title 17, U.S.C., §105 provides that copyright protection under this title is not available for any work of the U.S. Government. Title 17, U.S.C., §101 defines a U.S. Government work as a work prepared by a military Service member or employee of the U.S. Government as part of that person’s official duties.”

## References

[1] McCarthy JG, Warren SM, Bernstein J, Burnett W, Cunningham ML, Edmond JC, Figueroa AA, Kapp-Simon KA, Labow BI, Peterson-Falzone SJ, Proctor MR, Rubin MS, Sze RW, Yemen TA, Craniosynostosis Working G (2012). Parameters of care for craniosynostosis. Cleft Palate Craniofac J.

[2] Warren SM, Proctor MR, Bartlett SP, Blount JP, Buchman SR, Burnett W, Fearon JA, Keating R, Muraszko KM, Rogers GF, Rubin MS, McCarthy JG (2012). Parameters of care for craniosynostosis: craniofacial and neurologic surgery perspectives. Plast Reconstr Surg.

[3] Bonfield CM, Tamber MS, Losee JE (2014) Blindness from late presenting undiagnosed pancraniosynostosis mimicking pseudotumor cerebri. J Child Neurol 29:NP24–27. 10.1177/0883073813495307 10.1177/0883073813495307 23864590

[4] Fearon JA (2014). Evidence-based medicine: Craniosynostosis. Plast Reconstr Surg.

[5] Birgfeld CB, Dufton L, Naumann H, Hopper RA, Gruss JS, Haberkern CM, Speltz ML (2015). Safety of open cranial vault surgery for single-suture craniosynostosis: a case for the multidisciplinary team. J Craniofac Surg.

[6] Greives MR, Ware BW, Tian AG, Taylor JA, Pollack IF, Losee JE (2016). Complications in posterior cranial vault distraction. Ann Plast Surg.

[7] Morrison KA, Lee JC, Souweidane MM, Feldstein NA, Ascherman JA (2018). Twenty-year outcome experience with open craniosynostosis repairs: an analysis of reoperation and complication rates. Ann Plast Surg.

[8] Boulet SL, Rasmussen SA, Honein MA (2008). A population-based study of craniosynostosis in metropolitan Atlanta, 1989–2003. Am J Med Genet A.

[9] Jubbal KT, Agrawal N, Hollier LH (2017). Analysis of morbidity, readmission, and reoperation after craniosynostosis repair in children. J Craniofac Surg.

[10] Tahiri Y, Paliga JT, Wes AM, Whitaker LA, Bartlett SP, Taylor JA (2015). Perioperative complications associated with intracranial procedures in patients with nonsyndromic single-suture craniosynostosis. J Craniofac Surg.

[11] Kuo BJ, Vissoci JR, Egger JR, Smith ER, Grant GA, Haglund MM, Rice HE (2017). Perioperative outcomes for pediatric neurosurgical procedures: analysis of the National Surgical Quality Improvement Program-Pediatrics. J Neurosurg Pediatr.

[12] American College of Surgeons (2022) American College of Surgeons National Quality Improvement Program. Participant use data files 2012–2020. https://www.facs.org/quality-programs/data-and-registries/acs-nsqip/participant-use-data-file/. Accessed 20 Jul 2020

[13] Goobie SM, Zurakowski D, Proctor MR, Meara JG, Meier PM, Young VJ, Rogers GF (2015). Predictors of clinically significant postoperative events after open craniosynostosis surgery. Anesthesiology.

[14] Coombs DM, Knackstedt R, Patel N (2022) Optimizing blood loss and management in craniosynostosis surgery: a systematic review of outcomes over the last 40 years. Cleft Palate Craniofac J 10556656221116007. 10.1177/10556656221116007 10.1177/10556656221116007 35903885

[15] Hill GE, Frawley WH, Griffith KE, Forestner JE, Minei JP (2003). Allogeneic blood transfusion increases the risk of postoperative bacterial infection: a meta-analysis. J Trauma.

[16] O'Donnell DB, Vazquez S, Greisman JD, Uddin A, Graifman G, Dominguez JF, Zellner E, Muh CR (2022) Tranexamic acid dosing in craniosynostosis surgery: a systematic review with meta-analysis. Plast Reconstr Surg Glob Open 10:e4526. 10.1097/GOX.0000000000004526 10.1097/GOX.0000000000004526 PMC9575957 36262683

[17] Beethe AB, Spitznagel RA, Kugler JA, Goeller JK, Franzen MH, Hamlin RJ, Lockhart TJ, Lyden ER, Glogowski KR, LeRiger MM (2020) The road to transfusion-free craniosynostosis repair in children less than 24 months old: a quality improvement initiative. Pediatr Qual Saf 5:e331. 10.1097/pq9.0000000000000331 10.1097/pq9.0000000000000331 PMC7365706 32766502

[18] Najjar O, AbouChebel N, Zeeni C, Najjar MW (2021). Outcomes following craniosynostosis surgery at a tertiary care center in the Middle East. Pediatr Neurosurg.

[19] Shastin D, Peacock S, Guruswamy V, Kapetanstrataki M, Bonthron DT, Bellew M, Long V, Carter L, Smith I, Goodden J, Russell J, Liddington M, Chumas P (2017). A proposal for a new classification of complications in craniosynostosis surgery. J Neurosurg Pediatr.

[20] Lin Y, Pan IW, Mayer RR, Lam S (2015). Complications after craniosynostosis surgery: comparison of the 2012 Kids' Inpatient Database and Pediatric NSQIP Database. Neurosurg Focus.

[21] Bartz-Kurycki M, Wei S, Bernardi K, Moffitt JK, Greives MR (2019). Impact of cardiac risk factors on complications following cranial vault remodeling: analysis of the 2012 to 2016 National Safety Quality Improvement Program-Pediatric Database. J Craniofac Surg.

[22] Parikh RP, Farber SJ, Nguyen D, Skolnick GB, Patel K, Woo AS (2015). Risk factors for postoperative complications after surgical correction of craniosynostosis: a nationwide analysis of 1357 intracranial procedures. Plast Reconstr Surg.

[23] Xie C, De S, Selby A (2016). Management of the airway in Apert syndrome. J Craniofac Surg.

[24] Doerga PN, Spruijt B, Mathijssen IM, Wolvius EB, Joosten KF, van der Schroeff MP (2016). Upper airway endoscopy to optimize obstructive sleep apnea treatment in Apert and Crouzon syndromes. J Craniomaxillofac Surg.

[25] Papay FA, McCarthy VP, Eliachar I, Arnold J (2002). Laryngotracheal anomalies in children with craniofacial syndromes. J Craniofac Surg.

[26] Pereira V, Sacher P, Ryan M, Hayward R (2009). Dysphagia and nutrition problems in infants with apert syndrome. Cleft Palate Craniofac J.

[27] Sherrod BA, Johnston JM, Rocque BG (2016). Risk factors for unplanned readmission within 30 days after pediatric neurosurgery: a nationwide analysis of 9799 procedures from the American College of Surgeons National Surgical Quality Improvement Program. J Neurosurg Pediatr.

[28] Bruce WJ, Chang V, Joyce CJ, Cobb AN, Maduekwe UI, Patel PA (2018). Age at time of craniosynostosis repair predicts increased complication rate. Cleft Palate Craniofac J.

[29] Patel A, Yang JF, Hashim PW, Travieso R, Terner J, Mayes LC, Kanev P, Duncan C, Jane JJ, Jane JS, Pollack I, Losee JE, Bridgett DJ, Persing JA (2014). The impact of age at surgery on long-term neuropsychological outcomes in sagittal craniosynostosis. Plast Reconstr Surg.

[30] Bortz C, Pierce KE, Brown A, Alas H, Passfall L, Krol O, Kummer NA, Wang E, O'Connell B, Wang C, Vasquez-Montes D, Diebo BG, Neuman BJ, Gerling MC, Passias PG (2021) Frequency and Implications of Concurrent Complications Following Adult Spinal Deformity Corrective Surgery. Spine (Phila Pa 1976) 46:E1155-E1160. 10.1097/BRS.0000000000004064 10.1097/BRS.0000000000004064 34618707

[31] AMA CPT International (2023) CPT International. https://cpt-international.ama-assn.org. Accessed 28 May 2023

